# The Prevention and Treatment of Syphilis

**Published:** 1899-08-19

**Authors:** 


### THE PREVENTION AND TREATMENT OF SYPHILIS.

In the discussion at Portsmouth on the prevention and treatment of syphilis in the navy and army, while plenty was said to show that those who have studied the subject still look upon public measures of prevention, if properly carried out and universally applied, as furnishing the best means for controlling the ravages of this disease, it seemed to be pretty generally accepted that there was no present likelihood of such measures being enforced, and therefore attention was turned to other means of lessening the severity of the malady. It was generally recognised that one thing which is essential for the mitigation of the later effects of the disease, and for the prevention of those injuries which it now inflicts upon the innocent wives and children of its victims, is prolonged treatment; and, for the purpose of securing this, a good case seemed to be made out for the hypodermic administration of mercury. Major Lambkin, R.A.M.C., gave details in regard to intramuscular injection, and showed that by this method the treatment of syphilis could be maintained for as long a period as might be thought necessary with very little or no trouble to the patient. The following is the prescription which he has employed: Hydrarg., dr. 1; lanolin pur., dr. 2; ol. carbol. (1 in 20), dr. 4. Of this, 10 minims are to be injected into the buttock once a week, the whole course of treatment lasting about eight to twelve months. In 20,000 injections Major Lambkin had only once seen any bad results—a localised abscess at the point of injection. Other speakers gave testimony to the efficacy of the hypodermic method, and it was pointed out that in this way the collection of mercury injected might remain encapsulated for long periods, so that the patient carried his treatment with him for some years (!) While, however, it seemed to be admitted that public opinion was not likely at present to allow of any interference with the trade in vice which at present, with the full consent of our virtuous lawmakers, disgraces our streets, there was no hesitation in expressing the opinion that, so long as both law and custom give their sanction to the trade by which the disease is mainly propagated, the attempt to diminish the prevalence of the disease by other means is only likely to be attended with a very moderate degree of success. Indeed, it is but "making the best of a bad job."

